#  Physiological anthropometric associations with health metrics across different blood types among nursing students

**DOI:** 10.12669/pjms.41.12.13109

**Published:** 2025-12

**Authors:** Ayesha Sadiqa, Asma Khalid, Shaista Arshad Jarral

**Affiliations:** 1 Ayesha Sadiqa, Department of Physiology, Institute of Dentistry, CMH Lahore Medical College and Institute of Dentistry, Lahore, Pakistan. National University of Medical Sciences (NUMS), Rawalpindi, Pakistan; 2 Asma Khalid, Gulfreen Nursing College, Avicenna Medical, Dental College, Lahore, Pakistan; 3 Shaista Arshad Jarral, Department of Anatomy, Institute of Dentistry, CMH Lahore Medical College and Institute of Dentistry, Lahore, Pakistan. National University of Medical Sciences (NUMS), Rawalpindi, Pakistan

**Keywords:** Anthropometric measures, Blood pressure, Body mass index, Chest circumference, Glycemia, Mid-upper arm circumference, Pulse rate

## Abstract

**Objectives:**

To investigate the relationship between anthropometric measurements indices (chest and arm size) and health metrics (BMI, blood pressure, pulse rate, and glycemia) in healthy adults and to explore the influence of blood groups on these parameters.

**Methodology:**

A cross-sectional study was conducted with 157 nursing students of the University of Lahore from December 2022 to April 2023. In this study measurements of arm and chest circumference were taken from 157 nursing students. Blood pressure (BP) and pulse rate were measured per standard procedure. Blood typing was tested via agglutination, and glycemia was tested using biochemical assay techniques. Independent sample t-test and one-way ANOVA were applied to compare means between genders and blood groups respectively. Pearson-correlation was used for association of anthropometric measures with health metrics within each gender and blood group.

**Results:**

Participants reported a direct association between BMI and arm size (r=0.236, p=0.003), in A+ (r=0.296, p=0.043) and B+ blood (r=0.362, p=0.022); this association was significant. BMI was directly associated with chest size in total participants (r=0.299, p<0.001), males (r=0.618, p<0.001), females (r=0.231, p=0.012), A+ (r=0.387, p=0.007), and B+ blood (r=0.397, p=0.011). Chest size was directly associated with glycemia in females (r=0.220, p=0.017) and total participants (r=0.187, p=0.019); in males, arm size was directly associated with glycemia (r=0.400, p=0.012). Arm size was inversely associated with diastolic-BP in B-blood (r=-0.742, p=0.035). Chest size was inversely associated with diastolic-BP in O-blood (r=-0.710, p=0.014).

**Conclusion:**

Anthropometry and BMI were directly related, particularly in A+ and B+, suggesting these blood types are at higher risk for BMI-related cardiometabolic concerns. Males exhibited correlation of larger chest circumference with higher fasting glucose, indicating greater susceptibility to diabetes.

## INTRODUCTION

Studies consistently show a connection between BMI and various health conditions.^1^-^3^ However, the influence of other measurements, such as arm and chest circumference, is less well studied. Anthropometric measurements, including mid-upper arm circumference (MUAC), body mass index (BMI), waist-to-hip ratio (WHR), as well as vital signs and blood sugar levels (glycemia), are dimensions that have not been thoroughly explored when considering the relationship between blood types and these complex associations.^4^,^5^

MUAC is a more convenient measure for assessing an individual’s nutritional status. The reference values for anthropometric entities vary widely among various populations.^6^ The relationship between BMI and MUAC distinguishes between adults and children, as adults typically have higher BMIs.^7^ Elevated BMI, often associated with increased adiposity, has been linked to hypertension, altered heart rate, and disturbances in glucose metabolism.^8^ Monitoring blood glucose helps identify patterns of fluctuation in blood sugar levels triggered by various factors, including diet, physical activity, medication, and conditions such as diabetes mellitus. The normal range of glycemia is 72-108 mg/dL.^9^

Heart rate is one of the vital signs; in adults, the typical range is between 60 and 100 beats/minute, with tachycardia (more than 100 beats per minute) and bradycardia (less than 60 beats per minute).^10^ Studies have shown that obesity and overweight, often assessed using BMI, are associated with increased blood pressure.^10^,^11^ The relationship amongst chest size, arm size, and BP is less well-established.

ABO blood group antigens retain their immense vitality in the medical domain of blood transfusion because of their supreme capacity to cause antigenicity among all types of blood group agglutinogens.^12^ Comprehension of the ABO blood group not only significantly enhanced the safety of blood transfusions but also facilitated its utilization in forensic science and by anthropologists studying diverse populations.^13^ However, the role of all declared blood types in the relationship between health-related parameters and anthropomorphic features remains largely unexplored.

Recent research suggests that blood type may influence an individual’s susceptibility to certain health conditions.^14^ In the same connection, we aim to investigate the relationship between anthropometric measurements (chest and arm size) and health metrics (BMI, blood pressure, pulse rate, and glycemia) in healthy adults, and to explore the influence of blood groups on these parameters. That may help the clinicians to suggest more related laboratory investigations for certain blood types, due to their related high risk for cardiometabolic ailments e.g. diabetes, and hypertension. Clinicians may consider these correlations when assessing a patient’s metabolic risk variables to support individualized treatment plans.

## METHODOLOGY

A cross-sectional study was conducted with 157 nursing students of the University of Lahore in the 18-28-year age group, comprising 39 males and 118 females. The study participants were selected using a convenience sampling approach, and informed consent was obtained from each individual before data collection commenced. The study started in December 2022 and was completed in April 2023.

### Ethical considerations:

Sampling commenced following institutional ethical approval, received from the University of Lahore (REG/GRT/22/AHS-129; dated November 8, 2022). All students were guaranteed that their participation is voluntary and received assurances regarding anonymity and safety measures prior to data collection. No harm occurred during any phase of the sampling process.

### Inclusion and Exclusion criteria:

The present cross-sectional study included only physically healthy students who consented to participate, and it excluded those with infections, who used anticonvulsant drugs, or who had any other health concerns.

### Measurement protocols of anthropometric and clinical variables:

The participant’s height was measured in meters (nearest to 0.25 cm) using a plastic measuring ruler positioned vertically on the wall. The participant stood barefooted on the ground, while their head, shoulders, and buttocks touched the hard wall. The weight was measured using the least clothing using a weighing scale. The measurement of chest size was appreciated in inches through a flexible ruler (tape) at the mesosternal anatomical surface landmark on the front side of the human body, perpendicular to the thorax’s axis. The participant stood relaxed with arms slightly moved away for the tape placement, and the dimensions were recorded with the exhalation cessation. The ruler alignment was also ensured at the back side of the body.

The arm size (MUAC circumference) was also measured in inches (nearest to 0.1 cm) on the right arm using a flexible plastic measuring ruler placed equally between scapular and ulnar acromion processes. The heart rate (pulse frequency) was measured using the three-finger method. It was a manual method, where the subject was seated in a calm and relaxed state. The investigator gripped the subject’s radial artery over the right wrist with his (investigator’s) indexed finger and the middle finger was used to feel the heartbeat (pulse rate) with the second figure and counted the pulse rate for one minute (beats in one minute).

A standard protocol was followed to measure systolic and diastolic blood pressure (BP) by a single trained investigator each time using the standard routine clinical stethoscope and mercury sphygmomanometer. The subject was given 15 minutes of relaxation (in a calm sitting position on an armchair) before the BP measurement. Blood group identification was administered for Rh-type (anti-D related) and ABO blood types using the conventional method of agglutination. Here, the sample of blood was mixed with all three types of antisera, i.e., anti-D (Rh), anti-A, and anti-B, onto a glass plate. Then, samples were labeled based on erythrocyte agglutination. Glycemia, i.e., fasting blood sugar (FBS) levels, were estimated via biochemical testing of the collected blood samples after fasting for 8 hours in mg/dl.

### Statistics analysis:

The SPSS software was used to run all statistical tests. For descriptive-type analysis, averages and standard deviations deviation were calculated for anthropometric measures and health metrics. The imbalance between the two sexes was initially adjusted using the flexible application feature of the Bootstrapping technique in SPSS for statistical adjustment. An independent sample t-test was used for comparison of the averages between genders for each parameter, namely age, arm circumference, chest circumference, BMI, systolic BP, diastolic BP, pulse rate, and glycemia. For blood group comparisons, one-way ANOVA was used. The Pearson test of correlation was applied to confirm the existence of any relationship between anthropometric measures and health metrics. A 95% confidence internal was taken to establish statistical significance.

## RESULTS

Of 157 participants, the most frequently repeated blood type was A+, while AB- was the least frequent. Age-wise, no statistical difference was found between genders. A non-significant increase was noticed in the arm circumference of males, and a significant increase was observed in the chest circumference of males compared to females. Gender-wise, no significant change was reported in BMI, systolic BP, diastolic BP, or heart rate. However, glycemia was significantly higher in males (Table-I).

**Table-I:** Group-wise statistics related to each parameter of the study population.

Study Variables	Mean±S.D.		
	Total	Male	Female
Age (years)	22.31±1.83	22.43±1.55	22.27±1.19
	*P-value*	0.373	
Arm Circumference (inches)	10.60±2.97	11.61±2.52	10.27±3.04
	*P-value*	0.868	
Chest Circumference (inches)	29.54±7.06	33.15±3.06	28.34±7.52
	*P-value*	0.000*	
BMI (kg/m^2^)	20.58±3.98	21.44±4.71	20.29±3.68
	*P-value*	0.407	
B.P Systolic (mmHg)	110.45±9.88	115.64±7.17	108.73±10.08
	*P-value*	0.086	
B.P Diastolic (mmHg)	71.15±8.62	76.15±6.73	69.49±8.55
	*P-value*	0.250	
Pulse rate (beats/min)	82.21±10.16	80.00±9.43	82.94±10.33
	*P-value*	0.948	
Fasting Blood Sugar (mg/dl)	97.35±11.69	98.15±8.02	97.08±12.69
	*P-value*	0.001*	

* statistically significant (P-values are from t-test)

Although not significant, a positive association was observed between systolic BP and arm size. Similarly, an insignificant direct relationship was found between diastolic BP and arm size. A negative non-significant relationship was noted between upper-arm size and the heartbeat in males, females, and the total population. A direct significant relationship was found between arm size and glycemia in males. However, the same positive association was found to be non-significant in females and the total population. Statistically, a significant direct relationship was revealed between arm circumference and BMI in the total population and in the females but this positive association remained nonsignificant in the males (Table-II).

**Table-II:** Gender-wise Pearson’s Correlation of study variables in the population.

Categories	Arm Circumference : B.P Systolic		Arm Circumference : B.P Diastolic		Arm Circumference : Pulse rate		Arm Circumference : Fasting Blood Sugar		Arm Circumference : BMI		Chest Circumference : B.P Systolic		Chest Circumference : B.P Diastolic		Chest Circumference : Pulse rate		Chest Circumference :Fasting Blood Sugar		Chest Circumference :BMI	
	Pearson R	P-value	Pearson R	P-value	Pearson R	P-value	Pearson R	P-value	Pearson R	P-value	Pearson R	P-value	Pearson R	P-value	Pearson R	P-value	Pearson R	P-value	Pearson R	P-value
Total	0.088	0.272	0.099	0.220	-0.164	0.40	0.150	0.80	0.236	0.003*	0.071	0.376	0.128	0.110	-0.100	0.214	0.187	0.019*	0.299	0.000*
Male	0.109	0.539	0.159	0.335	-0.198	0.226	0.400	0.012*	0.278	0.086	-0.062	0.502	0.405	0.011*	-0.115	0.484	-0.175	0.287	0.618	0.000*
Female	0.016	0.073	0.010	0.918	-0.130	0.160	0.103	0.269	0.202	0.029*	0.362	0.023*	-0.013	0.891	-0.061	0.512	0.220	0.017*	0.231	0.012*

* statistically significant.

A significant positive association was found between BP (systolic) and chest size in women and the total population, but in males, this association was noted as negative and non-significant. A direct link was revealed between BP (diastolic) and chest size in the total population as well as in the males. However, this positive association was statistically non-significant in the total population, and significant in males. The same association was found to be negative and non-significant among females. The relationship between the size of chest and pulse rate was proven harmful and nonsignificant in study groups. There was a significant positive relationship between chest size and glycemia in the total study population and the females, though the same variables showed an inverse non-significant relationship. A strong direct relationship existed between size of chest and BMI in all three study groups i.e. total population, males, and females (Table-II).

No significant difference was observed among blood groups concerning each parameter. However, statistics showed that the maximum mean arm circumference was noted in B- group and the least in AB- group, while the mean maximum chest circumference was found in the A- group and the minimum was expressed again by the AB- group. The highest average BMI was observed in the AB- group and lowest in the B- group. The maximum mean systolic BP was noticed in the subjects with AB+ blood type and the minimum was found in those with A- blood type. Subjects with blood group B+ represented the highest mean diastolic BP, while those with A- blood group revealed the lowest mean diastolic BP. The maximum mean heart rate was found in O+ blood, while the least mean heart rate was observed in the blood type AB-, the same group expressed the highest mean glycemia and minimum mean glycemia was found in the O- blood group (Table-III).

**Table-III:** Statistical values for each parameter in the population based on their blood groups.

Blood Groups	Mean±S.D. of study parameters						
(n)	Arm Circumference (inches)	Chest Circumference (inches)	BMI (kg/m^2^)	B.P Systolic (mmHg)	B.P Diastolic (mmHg)	Pulse rate (beats/min)	Fasting Blood Sugar (mg/dl)
A+ (47)	10.68±2.63	29.17±7.64	21.22±4.24	110.43±10.62	71.70±10.06	81.81±10.48	95.70±12.22
A- (7)	11.28±1.97	33.28±1.25	18.71±2.71	105.71±12.72	67.14±9.51	77.85±6.59	103.28±7.45
B+ (40)	10.72±3.08	30.22±5.75	20.24±4.54	111.02±8.97	72.00±8.22	83.35±11.13	98.57±10.85
B- (8)	11.75±3.15	30.12±4.22	18.69±1.57	108.75±11.26	67.5±8.86	81.00±8.00	100.50±6.48
O+ (21)	9.45±3.14	30.31±8.70	21.35±3.72	110.00±10.48	71.90±6.01	86.62±10.42	95.81±12.09
O- (11)	10.38±3.98	27.61±6.74	20.28±3.59	107.27±9.04	68.18±7.50	78.63±9.85	94.09±15.52
AB+ (22)	10.91±3.01	28.25±8.25	20.49±3.62	113.64±8.47	71.81±8.5	80.72±8.54	98.00±11.90
AB- (01)	8.00	22.00	21.89	110.00	70.00	75.00	113.00
P-value	0.562	0.611	0.565	0.612	0.649	0.311	0.4518

P-values are from One-way-ANOVA.

When the association of arm circumference was assessed with systolic BP in each blood group, a statistically non-significant but positive association was observed in A+, A-, B+, O+, and AB+ blood, while a negative relationship was found in B- and O- blood. Similarly, when arm circumference was correlated with diastolic BP, only the B- blood group showed a significant negative association, while all other blood groups exposed a non-significant correlation. Though blood groups A+, A-, B+, O+, and AB+ revealed a positive correlation, blood group O- showed a negative association. Likewise, when all the blood groups were analyzed for their association between arm circumference and pulse rate, a nonsignificant positive association was noticed with B-, O+, and O- blood, while a nonsignificant negative association was observed in blood groups A+, A-, B+ and AB+. Only the AB+ blood type expressed a negative correlation between arm circumference and fasting blood glucose levels, for the same association, A+, A-, B+, B-, O+, and O- blood expressed an insignificant but direct relation. The association between arm circumference and BMI exposed a statistically significant direct relation in A+ and B+ blood and a nonsignificant positive association in A-, O+, O-, and AB+ blood, whereas only the B- blood expressed a nonsignificant negative association (Table-IV).

**Table-IV:** Pearson’s correlation of study variables in each blood group of the population.

Blood Group (n)	Arm Circumference : B.P Systolic		Arm Circumference : B.P Diastolic		Arm Circumference : Pulse rate		Arm Circumference : Fasting Blood Sugar		Arm Circumference : BMI		Chest Circumference : B.P Systolic		Chest Circumference : B.P Diastolic		Chest Circumference : Pulse rate		Chest Circumference :Fasting Blood Sugar		Chest Circumference :BMI	
	Pearson R	P-value	Pearson R	P-value	Pearson R	P-value	Pearson R	P-value	Pearson R	P-value	Pearson R	P-value	Pearson R	P-value	Pearson R	P-value	Pearson R	P-value	Pearson R	P-value
A+ (47)	0.137	0.359	0.135	0.365	-0.307	0.36	0.197	0.185	0.296	0.043*	0.232	0.116	0.259	0.079	0.011	0.943	0.202	0.174	0.387	0.007*
A- (7)	0.189	0.684	0.671	0.099	-0.73	0.867	0.186	0.690	0.574	0.178	-0.119	0.799	-0.060	0.898	0.086	0.854	-0.242	0.601	0.499	0.254
B+ (40)	0.173	0.286	0.24	0.134	-0.227	0.159	0.098	0.548	0.362	0.022	0.090	0.582	0.158	0.330	-0.220	0.173	0.247	0.125	0.397	0.011*
B- (8)	-0.131	0.757	-0.742	0.035*	0.397	0.331	0.021	0.961	-0.378	0.356	-0.387	0.344	0.277	0.507	0.030	0.945	-0.138	0.744	-0.090	0.832
O+ (21)	0.159	0.490	0.164	0.478	0.002	0.994	0.063	0.786	0.089	0.702	0.085	0.714	-0.150	0.516	-0.285	0.211	-0.134	0.562	0.218	0.342
**O- (11)**	-0.351	0.290	-0.369	0.264	0.251	0.456	0.512	0.107	0.202	0.551	-0.743	0.009	-0.710	0.014*	-0.159	0.640	0.269	0.424	0.133	0.696
**AB+ (22)**	0.116	0.607	0.294	0.184	-0.236	0.290	-0.072	0.751	0.338	0.124	0.211	0.346	0.338	0.124	-0.107	0.634	0.412	0.057	0.327	0.137
**AB- (01)**	-		-		-		-		-		-		-		-		-		-	

* statistically significant.

Relationship between BP (systolic) and size of chest expressed a significant negative relation in O- blood, while A- and B- blood also showed negative but nonsignificant association. A+, B+, O+, and AB+ blood revealed a positive association in the same regard. Similarly, only O- blood exhibited a significant negative relation between chest circumference and diastolic BP; blood groups A-, O+, and O- also showed a negative but non-significant association. For the same parameters, A+, B+, B-, and AB+ blood types expressed a positive though nonsignificant association. A nonsignificant relation was found between chest circumference and pulse rate in all the blood groups. However, A+, A-, and B+ blood revealed a positive relation, while B+, O+, O-, and AB+ blood types exposed a negative relation between these two parameters (Table-IV).

The statistical association between chest circumference and glycemia showed a significant positive relationship in AB+ blood, A+, B+, and O- blood types also expressed a positive but non-significant association. A-, B-, and O+ blood types for the same parameters showed a negative nonsignificant relation. When the association of chest circumference and BMI was statistically assessed in each blood group, a significant positive association was observed in blood groups A+ and B+, while all other blood groups expressed a nonsignificant association, where a positive association was found in blood groups A-, O+, O-, and AB+, while only the B- blood group showed a negative association (Table-IV).

## DISCUSSION

Our study found a positive association between men’s arm circumference and glycemia. A similar relation was found in another article in 2023, where anthropometrics was used as a diagnostic tool for diabetes mellitus. This study also showed that Mid-arm circumference is related to glycemia.^15^ Mid-arm circumference and BMI were also significantly related in our population. A study done in Africa also showed the same results in adult women^16^; however, this was non-significant in males in our study.

Our study also revealed a direct relation between BP (systolic) and chest size, just as Erdal did in the study in 2020.^17^ A positive relation was found in males for chest circumference and diastolic blood pressure, similar to a study in 2021 to see the relationship between BP and anthropometric indices. This study stated that in the male gender, BMI had the maximum association coefficient for systolic BP and that waist circumference was more related to diastolic rather than systolic BP.^18^

A positive correlation was seen between fasting blood glucose levels and chest circumference. Literature from all over the globe expressed the relationship of anthropomorphic features with insulin-resistant type-2 diabetes mellitus (DM). For example, epidemiological research conducted with Tehranian males and females of the adult age group, Iraqis, Taiwanese males, and Vietnamese proposed the notion that waist circumference can predict insulin-resistant DM equated with certain additional anthropometric measures.^19^

Identifying anthropometric parameters that predict the risk of cardiovascular diseases in adults battling obesity is essential for forging a path toward a healthier future. The identified significant direct link between BP (systolic) and chest size in women sheds light on the possible impact of anthropometric parameters on cardiovascular health. The 17% larger chest size in males than females is consistent with established physiological variations between genders. Gender dimorphism frequently changes body composition and distribution of lean and fat tissues.^20^

A significant positive association was found between the females’ chest circumference and systolic blood pressure. Our findings are consistent with earlier studies from multiple countries expressed a significant relationship between anthropometric dimensions and elevated BP (systolic).^21^,^22^ The complex relationship between chest circumference and systolic blood pressure involves regional adiposity, muscle mass, hormonal impacts, respiratory function, and genetic variables.^22^,^23^ Hormonal variations between males and females, such as estrogen and testosterone, may explain the observed gender-specific relationships. Estrogen has been connected with vasodilation, which could influence blood pressure regulation.^24^

In our study, males had 1.1% higher fasting blood glucose, consistent with earlier research indicating gender differences in glucose metabolism. The positive relationship between fasting blood glucose and arm size in males highlights the possible impact of regional adiposity on glucose regulation.^25^,^26^ Certain genetic markers have been associated with blood groups, and some research indicates that blood group antigens may be related to metabolic characteristics. Genetic factors influencing body composition may contribute to the observed positive association between arm circumference and BMI.^26^ Muscle mass and subcutaneous fat are included in the arm circumference measurement. BMI, a general measure of body composition, considers overall adiposity. In blood groups A+ and B+, genetic factors may influence the distribution of fat and muscle in a way that leads to a direct relationship between BMI and upper-arm size.^27^,^28^

In our current study, arm size demonstrated a negative correlation with the diastolic and systolic blood pressure in O-blood and B-blood. This suggests that variations in blood pressure regulation may be influenced by genetic variables linked to particular blood types, such as B- and O-. The antigens present in the blood possess a prime role in vascular function and responsiveness, leading to negative associations with arm size. These antigens could affect endothelial function and vasomotor tone, thus contributing to the observed negative associations with arm size.^28^

### Study limitation and Future Directions:

Non-significant associations in certain blood groups, such as A-, O+, O-, and AB+, may be attributed to a limited sample size, reducing statistical power. Larger cohorts may reveal more significant associations. Recognizing the gender-specific and blood type-specific relationships between metabolic markers and anthropometric variables is essential for customized health evaluations.

## CONCLUSION

Anthropometric measures, particularly arm and chest circumference, showed significant correlations with BMI, blood pressure, pulse rate, and glycemia across various blood groups. Increased BMI was consistently linked with higher chances of developing hypertension, cardiovascular disorders, and diabetes. Notably, in A+ and B+ blood groups, BMI correlated with chest size, and increasing arm and chest size was linked to higher glycemia, especially in males. Inverse relationships between arm size and diastolic BP in the B-blood group and chest size in the O-blood group suggest a potentially lower cardiovascular risk. Males exhibited significant correlation of larger chest circumference with higher fasting glucose, indicating greater susceptibility to diabetes than females.

### Authors’ Contribution:

**AS:** Conceived, designed, and did statistical analysis & editing of manuscript, is responsible for the integrity of the research.

**AK:** Did the data collection and manuscript writing.

**SAJ:** Interpreted the data, analyzed it, and wrote sections of the manuscript.

**AS, AK and SAJ:** Did the review and final approval of the manuscript.
